# Agricultural co-operatives participating in supply chain integration in China: A qualitative comparative analysis

**DOI:** 10.1371/journal.pone.0250018

**Published:** 2021-04-28

**Authors:** Jiacheng Zhang, Jianli Luo, Jia Li

**Affiliations:** 1 Business School, Wenzhou University, Wenzhou, Zhejiang, China; 2 School of Finance and Economics, Zhejiang Dongfang Polytechnics, Wenzhou, Zhejiang, China; Szechenyi Istvan University, HUNGARY

## Abstract

Agro-food supply chain integration (ASCI) plays a growingly important role in the stable and sustainable development of agriculture. However, it is challenging for core firms to integrate the small-scale and scatted farmers due to complex transaction processes and volatile relationships in China. Agricultural co-operatives are organizations that unite farmers’ power and help them achieve economic benefits. Our research focuses on ASCI from the perspective of co-operatives. A comprehensive cooperative framework, including trinity co-operatives and trinity federations, is conducted to figure out the position and process of agricultural co-operatives in ASCI, while QCA provides detailed collaborative patterns for agricultural co-operatives to adopt. Results show that agricultural co-operatives can achieve high economic and social/environmental performance when participating in ASCI. This study further completes the ASCI literature and offers many managerial and academic implications to co-operatives’ members and policy-makers.

## 1 Introduction

ASCI plays a crucial role in the stable and sustainable development of agriculture. Efficient ASCI can achieve high performance, such as increasing sales, ensuring food safety, improving product quality, enhancing food traceability, and reducing food corruption [1–3]. However, traditional small-scale farmers in China have many problems, such as backward technology, scattered land, and lack of all kinds of resources [4]. These problems lead to cumbersome trading processes, exorbitant trading costs, and unstable relationships in ASCI, making it more challenging for core firms to effectively integrate the small-scale and scatted farmers and control the quality of production [5–7]. To solve these problems, this study turned attention toward the agricultural co-operatives in China and discusses their participation in ASCI by analyzing their organizational advantages and unique features.

According to the international Cooperative Alliance [8], co-operatives are defined as villagers’ autonomous organizations leagued by farmers’ willingness for the purpose of achieving economic and social needs. The establishment of co-operatives aims to realize economic or social benefits for farmers, enhancing farmers’ bargaining power in the market, reducing trading costs, and centralizing funds and information [9]. The development of agricultural co-operatives, which is encouraged in almost all countries, has long been an explicit goal of public policy. In many European countries such as Spain and the United Kingdom, governments and agricultural institutions have paid increasing attention to co-operatives’ position and role in the food value chain in recent decades [10]. According to the data from ICA [11], the vast majority of farmers in many countries are members of agricultural co-operatives. They face the challenges in ASCI by joining co-operatives to gain economies of scale and scope, high-tech technologies and financial support [12]. The study of Istudor, Ion [13] and Mirel, Florentin [14] points out that agricultural co-operatives can improve farmers’ status in the supply chain, increasing product sales, reducing farmers’ costs, and even playing a positive role in protecting the environment. In the relevant studies in South Korea [15], China [16] and other Asian countries, we also find that people lay more and more emphasis on how to connect agricultural co-operatives with other individuals in ASC and attach great importance to the economic and social benefits they may bring. From the concepts and characteristics above, we can find that the agricultural co-operative is likely to be an active part of ASCI.

So far, most articles have discussed how can companies participate in supply chain management, and the literature on ASCI mostly focus on its structure and overall effects. Hence, we have little understanding of co-operatives’ role in ASCI in developing countries. In this article, we are going to conduct a further study on agricultural co-operatives for their participation in ASCI in China.

This research has certain theoretical and practical significance. First, our research contributes to the ASCI literature from the perspective of the latest trinity agricultural co-operatives in terms of production cooperation, supply & sale cooperation, and credit cooperation, finding them capable of completing internal and external integration. Second, this work is the first study to explicitly explore the general co-operative framework for trinity agricultural co-operatives in ASCI and figure out what can co-operatives achieve (especially the social/environmental performance) in detail after taking part in ASCI. Finally, based on QCA, we propose six collaborative patterns for agricultural co-operatives to adopt.

## 2 Literature review and research questions

### 2.1 Agro-food supply chain integration (ASCI)

ASCI binds the conventional concepts of Agro-food Supply Chain Management [17] and Supply Chain Management [18, 19] together. It can be defined as the degree to which a specific organization in Agro-food supply chain works effectively with upstream and downstream partners, completing internal and external integration in a farm-to-table sequence that consists of production, supply, marketing, carriage, and so on.

Internal integration for a specific organization refers to the synergies across departments for information, data and resources to meet consumer demands and improve product quality with low costs in the material purchase, technology upgrade, market operation, and logistics transportation, etc. [16, 20–22]. External integration (i.e., supplier and customer integration) refers to the inter-organization systematic and forward-looking coordination between a specific agricultural organization and its ASC partners, such as suppliers, customers, and others. Such integration usually results in the synchronous practice of information sharing, strategic sales alliances, consumer demand forecasting, and establishing strategic partnerships with suppliers, customers, logistics and other relevant external partners to achieve collaborative work and generate typical value [23, 24].

The objectives of ASCI are to achieve the fast and efficacious flow of materials, information, data, and other different resources, ultimately reducing non-essential consumption, improving product quality, meeting market needs and preventing environmental damage [17, 19, 25, 26]. Although ASCI has such substantial advantages, it has not been studied carefully, and there is no theoretical model in the current literature for agricultural co-operatives to participating in ASCI. Additionally, challenges exist in China in ASIC, such as the small-scale of numerous supply chain entities, low level of technicalization and informatization, insufficient observation of market, and under-prepared cold chain logistics system [5, 6]. All these difficulties that China faces make the ASCI even more challenging.

### 2.2 Agricultural co-operatives in China

Many researchers and practitioners have paid attention to the importance of agricultural co-operatives in rural areas in boosting economic development. Under the condition of the small-scale agricultural economy in China [4], it is challenging to achieve intensification, specialization, and industrialization through single-family farming. Therefore, by uniting farmers, Chinese traditional agricultural co-operatives gain advantages in the system and break the scattered and unconsolidated weakness of family farming [7, 27]. Since the promulgation of the Farmers’ Professional Co-operatives Law in 2007, Chinese agricultural co-operatives have entered a stage of rapid development. Many co-operatives play a leading role in technology transfer, harmful agricultural pollution reduction, and sustainable agricultural economy development in China [28]. By the end of July 2020, 2.207 million farmer co-operatives had been registered nationwide [29]. Although traditional co-operatives have achieved a certain degree of scale and industrialization, they have disadvantages in cooperation with external partners.

In order to solve the difficulties existing in negotiation with the external companies, a new type of agricultural co-operative called the Rural Trinity Co-operative (i.e., the Trinity Co-operative) was proposed [28]. It carries out cooperation in production, supply & sale, and credit, receiving macro guidance from the corresponding Trinity Federation of Farmer’s Co-operative Economic Organization (i.e., the Trinity Federation), which also has three components of production, supply & sale, and credit [4]. They are the latest agricultural co-operatives in China that develop and expand from Zhejiang Province, gradually spreading to the whole country in 2018 [4, 6].

Compared with large-scale and high-tech co-operatives in developed regions, especially some transnational co-operatives in Europe [30], agricultural co-operatives in China are still in the state of fragmentation with low integration degree and deficient competitiveness [31]. The emergence of the trinity co-operatives and the trinity federations gives us some reason to believe that it is possible for these latest Chinese agricultural co-operatives to gain achievements in technological innovation, product supply and marketing, agricultural fund guarantee, and to improve the integration degree of ASC.

### 2.3 Trust in co-operatives

A crucial role of co-operatives is the realization of farmers’ participation, commitment and trust through the establishment of membership policies [32]. Farmers’ preference to sell their agricultural products to co-operatives rather than to other individuals stems from their trust and commitment. Many scholars classify trust into various types, such as cognitive trust and affective trust [33, 34]. Vasa, Hansen, Baranyai et al. also point out that trust can be classified according to different levels (e.g., trust between two members, trust among multiple members, and trust between members and managers) [34, 35].

Trust is an important factor for co-operatives’ cohesion and competitiveness [34]. Peng et al. [32] believe that communication and trust play a leading role in farmers’ participation in agricultural co-operatives in transition countries and the mutual trust of members is the foundation for co-operatives’ efficient operation. The results by Hansen et al. suggest that the degree of mutual trust among co-operative members and managers is a good indicator of group cohesion, which measures members’ willingness and commitment to stay in the co-operatives [35]. They also find that trust among members is more vital than trust between members and managers in terms of team cohesion and member satisfaction.

In ASCI, trust is also a topic worth discussing. Communication and trust between co-operatives and other agricultural supply chain partners can improve farm profitability and reduce production costs [34]. After studying the extensive literature on agricultural economics, Sodano [36] emphasizes that trust is the key to the success of co-operative relationship and she also points out that trust can enhance integration and coordination within co-operatives as well as promote cooperation between co-operatives and other supply chain partners.

### 2.4 Gap in literature

Collating the relevant studies, we find that most of them discuss the problems of companies’ participation in the SCI [20]. However, few scholars have conducted detailed researches on the co-operatives’ roles in ASCI, let alone the studies especially concentrating on the supply chain integration models from the perspective of agricultural co-operatives. Although agricultural co-operatives share some common features with traditional firms, they differ significantly in their objectives, governance and composition [31, 37] and these make co-operatives’ participation in ASCI become a gap in the literature. Besides, since the latest agricultural co-operatives (i.e., trinity co-operatives and trinity federations) have just entered the promotion stage in China, the empirical research on them is still rare.

This study tries to bridge the gap by discussing the possibility for agricultural co-operatives to participate in ASCI and proposing a general cooperation framework that consists of all related partners like trinity co-operatives, trinity federations, agricultural suppliers, customers, agrarian service centers, credit guarantee companies, and other service agencies as well as figuring out the collaborative patterns of agricultural co-operatives. To sum up, this research attempts to solve the following problems:

Q1. Why is it possible for agricultural co-operatives to participate in ASCI?
Q2. What is the framework for agricultural co-operatives in ASCI?
Q3. How should agricultural co-operatives act specifically in ASCI?

## 3 Models for agricultural co-operatives participating in ASCI

In this section, we firstly answer Question 1 by analyzing the nature and structure of the latest agricultural co-operatives (i.e., trinity co-operatives and trinity federations). From the perspective of internal and external integration, we find that agricultural co-operatives are capable of participating in ASCI. Secondly, we build a comprehensive cooperation framework model to figure out the position and process of agricultural co-operatives in ASCI (Question 2). Third and finally, we provide collaborative patterns for agricultural co-operatives to answer Question 3 and discuss them in detail in Section 4.

### 3.1 Internal integration of trinity co-operatives in ASC

**Fig 1:**
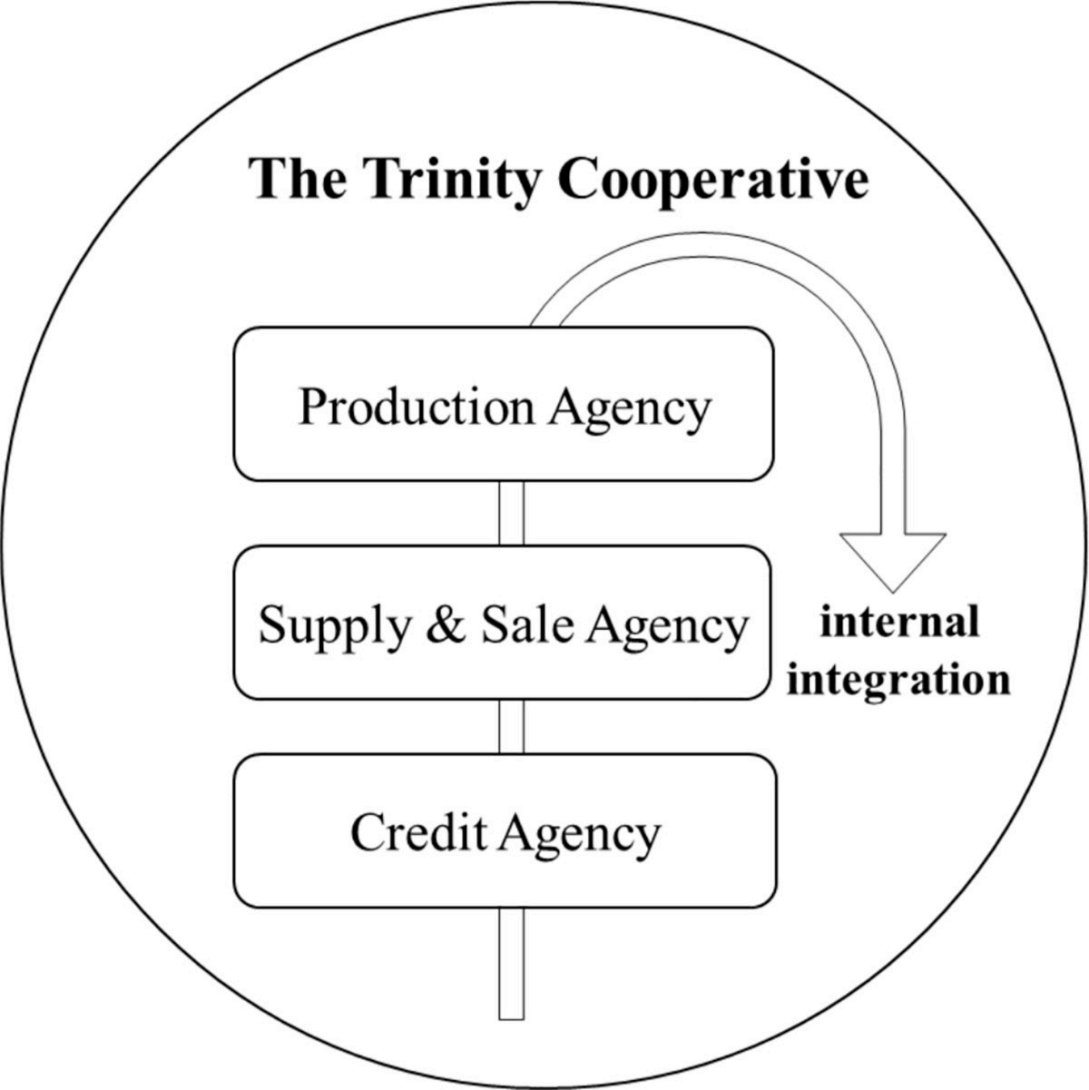
The internal integration of the trinity co-operative.

Compared with ordinary companies, the degree of information asymmetry of co-operatives is relatively low, because the producers participate in the governance of the organizations to some extent [38]. The collective and autonomous nature, members’ commitment, and mutual trust can strengthen the synergy for co-operative’s information, processes, and marketing across functions and departments by close contact and information exchange among co-operative members [39]. The interdepartmental information interaction can be seen as the internal integration of ASCI (see Fig 1). Within the trinity co-operative, various service platforms for the production, supply & sale, and credit are set up by farmers themselves voluntarily [28], which leads to a more close exchange of information among different departments. Internal supply chain integration makes the trinity co-operative into a cohesive whole, creating competitive advantages, improving working performance, and laying the foundation for the integration of the external supply chain.

What have been discussed above are the characteristics of typical co-operatives. In real life, some capable trinity co-operatives not only work internally but also cooperate with agricultural suppliers and customers to achieve external integration [4]. Most of these trinity co-operatives have the high-tech or patents of some agricultural products and their commodities gain enormous popularity in the local market. For example, The Huangzhang-Planting Agricultural Co-operative (Chuzhou, Anhui Province, China), has occupied the local market with its new varieties of chrysanthemum tea and established a stable relationship with local supermarkets and agricultural products stores. Cases of using new technology to achieve external integration can also be found in the aquaculture co-operatives, such as the Hezhou Turtle Co-operative (Changde, Hunan Province, China). These phenomena are more common in some transnational agricultural co-operatives, such as Arla Foods from Europe, whose market position and competitiveness are strong enough to influence other individuals and competitors in ASC [30, 40].

Trust is an indispensable part in the internal integration of co-operatives. In many Chinese co-operatives (e.g., trinity co-operatives), trust among members improves the authenticity and efficiency of information exchange. For example, in the Meiyu trinity co-operative (Wenzhou, Zhejiang Province), members participate in the operation of the co-operative through relevant rules directly or indirectly. Reasonable regulations and close interpersonal relationship enable co-operative members to have good cognitive trust and affective trust. These advantages will facilitate the daily communication and information exchange in agricultural co-operatives, and boost co-operatives’ efficiency and competitiveness.

### 3.2 External integration of trinity federations in ASC

**Fig 2:**
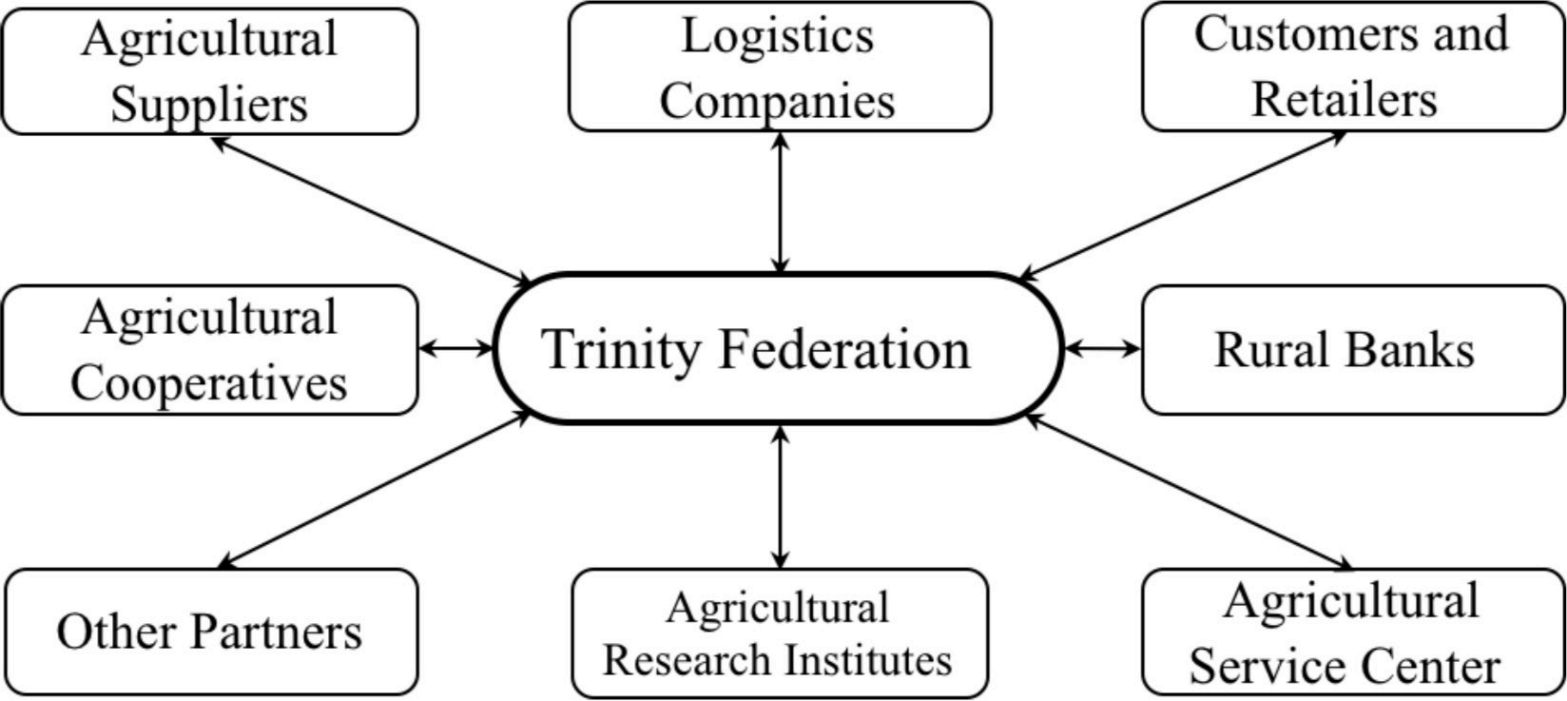
The external integration of the trinity federation.

The trinity federation is an organization administered by local governments, reforming the traditional supply & sale co-operative to the levels of province, city, county and town based on a macro view [28]. From the macro perspective, trinity federations can use the government power to cooperate with the supply chain patterns like agricultural retailers, research institutes, credit guarantee companies, and rural banks, increasing the negotiation ability of co-operatives. Evidence from Malaysia [41], Denmark and South Korea [15] also shows that the externalities of agricultural co-operatives in ASCI can bring economic benefits to themselves and other organizations. This kind of inter-organization strategic coordination between trinity federations and their partners could be considered as the external integration of ASC (see Fig 2) which could contribute to practical synergies of information exchange, marketing cooperation, material planning, resource sharing, and establishing a stable relationship with upstream and downstream of the supply chain.

Trust also plays a significant role in trinity federations’ external integration. Sodano ’s research shows that trust is the basis for the establishment of a stable and efficient agricultural supply chain [36]. Trust in ASCI can greatly reduce the workload between nodes and cut the transaction cost in supply chain. Qinhuangdao trinity federation (Qinhuangdao, Hebei Province) is a good example of this. This federation takes advantage of its long-term trusting relationship with local agricultural suppliers, vegetable markets and groceries to promote the stable cooperation with supply chain partners and improve the response capacity of the whole chain.

### 3.3 ASCI framework of agricultural co-operatives in China

#### 3.3.1 ASCI of trinity co-operatives and federations

**Fig 3:**
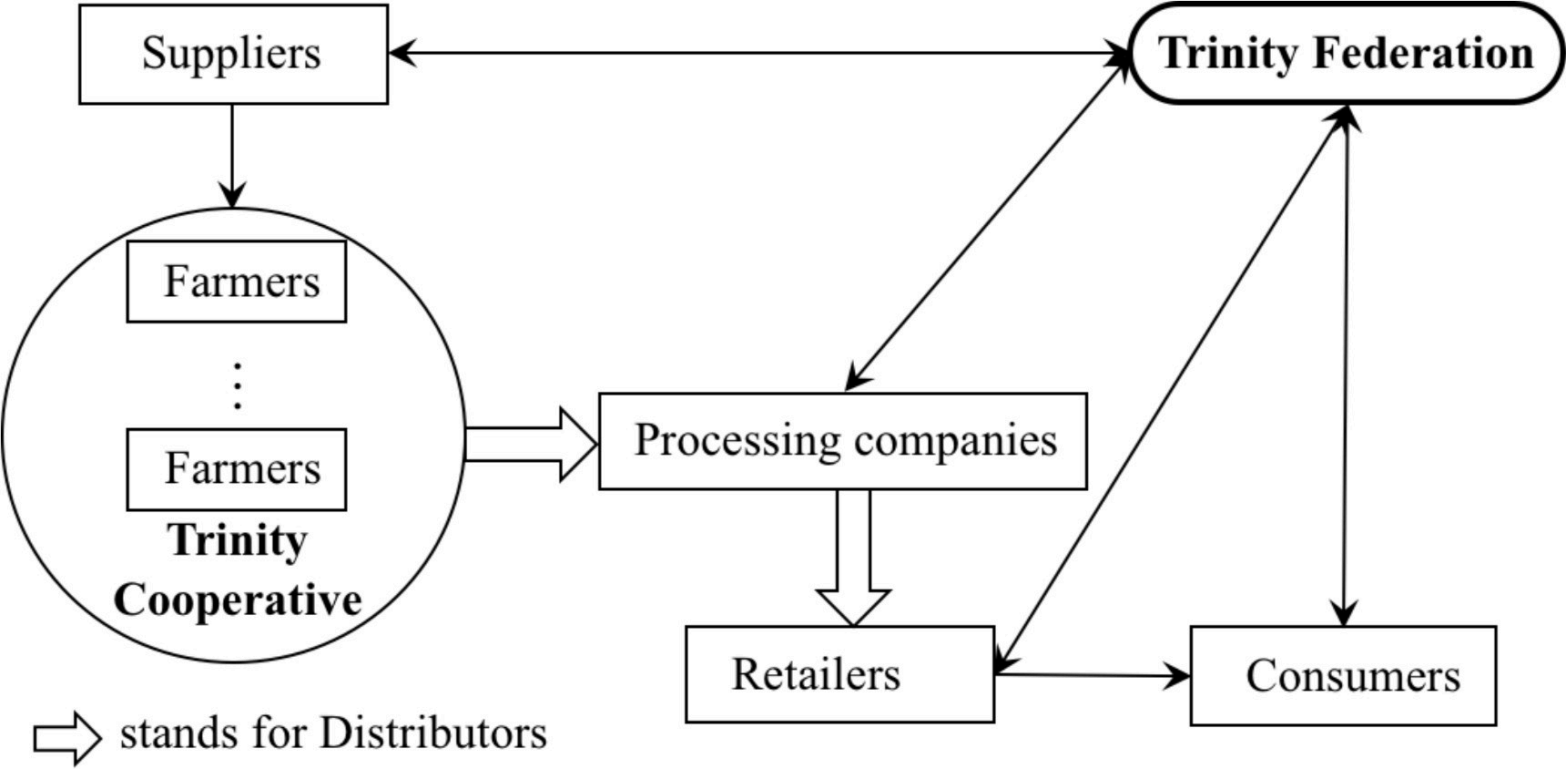
Framework for agricultural co-operatives in ASCI.

After analyzing the organizational structure of the comprehensive co-operatives (including trinity co-operatives and their corresponding trinity federations), we can find that this kind of co-operatives can complete internal and external integration through their organizational composition. When we add the existence of agricultural co-operatives into the ASC, we will get the framework (see Fig 3).

As shown in Fig 3, the trinity federation can introduce high external technologies to increase the productivity of agricultural products, and some can even improve the ability of technology innovation and patent acquisition [28, 31]. When the trinity federation takes the lead in production cooperation, it can set up Agricultural Service Center, Technology Innovation Center, and Agricultural Residue Testing Center with the help of agricultural research institutes, promoting the use of fertilizers and pesticides, improving quality control of agriculture production. Also, the trinity federation’s participation in sales extension and marketing can enhance the efficiency of expansion and reduce sale cost. Depending on its government’s power, the trinity federation can set up Service Center to connect the suppliers and retailers, helping farmers buy agricultural materials at a lower price, cutting down procurement costs, and guaranteeing the food safety. This method of using co-operatives and other relevant organizations to gather farmers’ power and strengthen their status in ASC can be seen in Europe [42], Asia [16] and many other places.

In terms of internal integration within agricultural co-operatives, trinity co-operatives understand farmers’ technology and production requirements since the close ties between co-operative members. They use this knowledge to accelerate the exchange of information flow on production, supply & sale, and credit [4, 28]. There is a specific case of internal integration in ASCI by a trinity co-operative. In the case of the Meiyu trinity co-operative (Wenzhou, Zhejiang Province), the integration of the interdepartmental information, prediction of future productivity and market direction has significantly improved the production and marketing capacity of the co-operative. The Meiyu trinity co-operative has specifically built four new greenhouse vegetable demonstration bases for its best-selling agricultural products through the collection of previous sales data. Agricultural loans are also being used to expand the production of tomato variety FA-189, which is the most competitive products predicted by information exchange of the production department and sales department in the co-operative. Additionally, the Meiyu co-operative has created many famous high-quality food brands such as Qianglv and Lv-yinxiang that are in high demand in the local vegetable market. The case of Meiyu demonstrates the importance of internal integration by a trinity co-operative in ASCI.

Although financial integration is not mentioned in traditional ASCI theory, many current theories in China [28], Spanish [43], the US [44] and other countries point to it that funding and insurance services have a remarkable effect on production and marketing. The credit cooperation of trinity co-operatives and trinity federations guarantees the financial support for using modern technologies, establishing new research bases, and conducting other projects through mutual insurance and rural banks. In addition, the efficient logistics company is an easily neglected part in ASCI (represented by the hollow arrow in Fig 3). Agricultural production is strongly influenced by the weather, and crops will easily decay if there is no cold chain delivery in a timely manner in the market.

Social/environmental performance is also a factor to be considered when discussing ASCI. In 2017 alone, 420 major agricultural food recalls were issued by the U.S. Food and Drug Administration [45], covering almost every category. Many food safety experts believe that the lack of information exchange among members of ASCI is one of the leading causes of today’s food safety problems [46]. Food quality issues are not easy to resolve for many reasons, including the difficulties of handling food quality in terms of appearance, insufficient information exchange in the farm-to-table sequence, and the conflicts between high quality and low price [5]. Facing these challenges, trinity federations and trinity co-operatives can well play their roles in ASCI. On the issue of the supply chain information asymmetry, trinity federations will improve the quality of agricultural products by trading with agricultural material suppliers for new seeds and low pollution fertilizers. Also, they can reduce the waste of agricultural products in cooperation with efficient logistics companies. Trinity co-operatives will steadily accelerate internal information exchange to adapt to market changes, pursuing organic green products to meet consumer demands. Through the framework above, agricultural co-operatives not only can achieve high economic performance such as expanding sales, reducing costs and increasing profits, but also can achieve social/environmental performance such as ensuring food safety, optimizing agricultural product structure and reducing spoilage, which plays an active role in the sustainable development of agriculture and the promotion of environmental improvement.

#### 3.3.2 Collaborative patterns of trinity co-operatives and federations in ASCI

So far, we have answered Questions 1 and 2, but Question 3 (how should agricultural co-operatives act specifically in ASCI?) remains unresolved. Zheng and Luo [47] pointed out that the repeated function, conflict of related departments, ineffective inter-organizational communication, and over-complicated institutional setup in agricultural co-operatives will reduce the co-operative’s performance to some extent and bring unexpected harmful effects. It means that the internal information integration in terms of production, supply & sale, and credit carried out by trinity co-operatives as well as the external integration in terms of production, supply & sale, and credit carried out by trinity federations may not all be necessary. Therefore, which combination of these six elements can achieve high performance is the focus of this section. (i.e., what kind of collaborative patterns should be adopted between trinity co-operatives and trinity federations).

**Fig 4:**
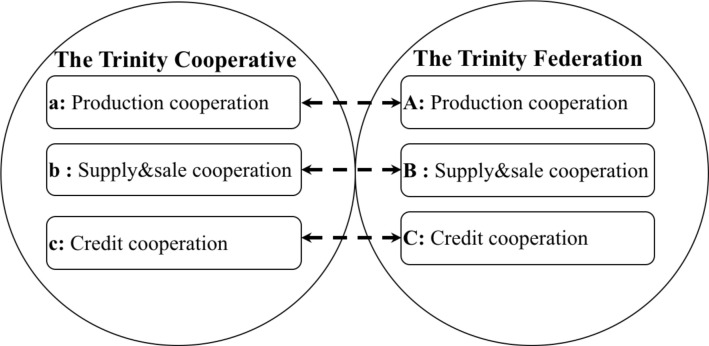
The cooperation patterns of trinity co-operatives and federations.

The collaborative patterns can be represented in Fig 4. The trinity federations and the trinity co-operatives both have functions in production, supply & sale, and credit. For simple expression, we use (A, B, C) to represent trinity federation’s (production, supply & sale, credit) cooperation, (a, b, c) to represent trinity co-operative’s (production, supply & sale, credit) cooperation, and (~) to represent the absence of a specific situation (the complement of the original set). According to the concept in QCA [48], we can get 64 possible primitive expressions between the trinity federations and the trinity co-operatives in parts of production, supply & sale and credit cooperation (64 Conditions/Truth table rows in total), but which ones are effective in the ASCI? This question is not mentioned in previous literature and is also the most critical part of this study, which will be discussed in the following sections.

## 4 The empirical analysis

### 4.1 QCA methodology

Considering the initial stage of the researches on latest agricultural co-operatives in China (i.e., the trinity co-operatives and the trinity federations) and the difficulties in collecting data through a wide range of survey methods, we adopted a qualitative comparative analysis (QCA). However, the central argument in our research to use QCA cannot be the number of cases in the investigation, but should be driven by the theories and expectations and what the researchers are interested in [49].

Combined what we want to study and its logical form, we have good reasons to believe that the factors for high performance of co-operatives should be explained as the result of causal complexities in terms of equifinality, conjunctural causation, and asymmetry in QCA [48]. There are more than one paths leading to the high performance of the agricultural co-operatives in real life, which is a good representation of equifinality. Furthermore, the cooperation factors of product, supply & sale, and credit in the trinity co-operatives and trinity federations may have an aggregation effect. Their impacts on the outcome have to be combined and that is what conjunctural causation draws attention to in QCA [49]. Moreover, asymmetrical causation implies that the presence or absence of every single condition in agricultural co-operatives may have a critical impact on the outcome. In all, we have every reason to believe that QCA is a suitable logical choice for this study.

### 4.2 Data collection

The analysis samples of this study are the trinity co-operatives and trinity federations, and the research purpose is to find the effective collaborative patterns of them. Through secondary data collection and field visits, 65 samples were selected as alternative case sets. The selection criteria are as follows:(1) The selected cases must be the latest agricultural co-operatives (i.e., the trinity co-operative and the corresponding trinity federation). (2) The chosen cases must have three kinds of cooperation in terms of production, supply & sale, and credit. (3) The selected cases must be representative and able to cover different regions. After further screening, 26 samples were finally selected.

### 4.3 Data analysis

#### 4.3.1 Set membership scores

The underlying principle of the QCA is Boolean algebra [48], and thus the raw data must be transformed into fuzzy sets (i.e., set membership scores) ranging from zero (full exclusion from a set) to one (full inclusion). It is critical to assign set membership scores in QCA [50, 51], and the process of using empirical information on cases for assignment is called “Calibration.”

#### 4.3.2 Calibration of outcome variables

The co-operatives participating in ASCI effectively will probably achieve “high economic performance” or “high social/environmental performance” in their operations. If the agricultural co-operatives can take part in supply chain integration, it is expected that they can have easier accesses to new agricultural resources, expanding sales of agricultural products, able to negotiate with other agricultural material or logistics companies and achieving effective exchange of internal information [25, 28, 52]. Thus, it can be speculated that these co-operatives generally have high profits (high economic performance). On the other hand, some co-operatives are in the primary stage and the benefits are not so obvious for the time being, but the agricultural products they produce could also be of high quality that can ensure food safety, optimize agricultural product structure, reduce spoilage and will drive surrounding farmers (high social/environmental performance). Therefore, economic performance and social/environmental performance are used to measure outcome variables in this study.

The set membership scores of outcome values (i.e., the performance of trinity co-operatives and federations) are calibrated as 0, 0.33, 0.67, 1 by the calibration criteria (see Table 1). The overall profits of the co-operatives and the average incomes of the co-operatives’ members are used to describe the economic performance. The quality of the products and the scales of the co-operatives are used to describe the social/environmental performance.

**Table 1:** Calibration criteria of the outcome variables.

Set membership scores	Calibration criteria of economic performance	Calibration criteria of social/environmental performance
1.00	the average annual revenue of the co-operative and its farmers’ incomes increased by more than 40%	the co-operative’s products are organic, its scale is vast, and its social reputation is good
0.67	the average annual revenue of the co-operative and its farmers’ incomes increased by 20–40%	the co-operative’s products are green, and it attracts a lot of surrounding farmers
0.33	the average annual revenue of the co-operative and its farmers’ incomes increased by 5–20%	the co-operative’s products are pollution-free, and it attracts a part of surrounding farmers
0	the average annual revenue of the co-operative and its farmers’ incomes increased by less than 5%	the co-operative’s products are of average quality, and it attracts few surrounding farmers

#### 4.3.3 Calibration of condition variables

In order to be analytically fruitful, Calibration of condition variables follows:

(a) a careful definition of the relevant latest agricultural co-operatives (i.e., trinity federations and trinity co-operatives); (b) a careful definition of all the concepts used in the analysis; (c) a careful decision on where the points of the maximum indifference about membership (i.e., the 0.5 anchor), the full membership (1) and the full non- membership (0) are located.

As mentioned above, to calibrate the set membership scores of the condition variables, three anchor points need to be preset: full membership, full non-membership, and a crossover point. Referring to the researches of Schneider and Wagemann [48], the paper sets the upper quartile, average value, lower quartile of the sample data as three anchor points of the six target sets (the target sets are a, b, c, A, B, C).

Since the cooperation of trinity federations and co-operatives are relatively complex concepts, this study subdivides each condition variable into the following second-level condition variables. According to the actual importance degree, the second-level variables are graded by 0–1 (i.e., the weight), and the six first-level condition variables can be obtained by multiple second-level variables. The second-level variables and their weights are shown in Table 2.

**Table 2:** Second-level condition variables and their weights.

Aspect of co-operation	Trinity co-operative	Weight	Trinity federation	weight
**Production cooperation**	Technology department	0.4	Technical popularization	0.3
	Production department	0.3	Labor service	0.3
	Purchasing and processing	0.3	Unified degree	0.2
	/	/	Processing service	0.2
**Supply & sale** **cooperation**	supply department	0.4	Agricultural material supply & sale service	0.3
	Product marketing department	0.4	Logistics and transportation service	0.2
	Circulation of goods	0.2	Brand and trademark service	0.2
	/	/	Purchasing and selling agency	0.3
**Credit cooperation**	Financial department	0.4	Financial support	0.4
	Insurance support	0.3	Credit Support	0.3

According to the weights of the second-level condition variables mentioned above, we can get the calibration criteria/thresholds of the condition variables (Table 3).

**Table 3:** Calibration criteria/thresholds of condition variables.

condition variables	Full non-membership	Crossover point	Full membership
*a*	0.37	0.50	0.64
*b*	0.24	0.33	0.40
*c*	0.27	0.34	0.38
*A*	0.37	0.59	0.60
*B*	0.13	0.25	0.35
*C*	0.08	0.12	0.20

### 4.4 Results

#### 4.4.1 The analysis of necessity

In QCA, the necessary condition refers to the condition that must appear in all the outcomes [48, 49]. In this study, it means the condition that must appear in outcomes of “high economic performance” and “high social/environmental performance”. FsQCA3.0 software was used to analyze the necessary condition and the specific results are shown in Table 4.

**Table 4:** Necessary condition.

condition variables	economic performance		social/environmental performance	
	Consistency	Coverage	Consistency	Coverage
*a*	0.658903	0.613716	0.681137	0.603919
*b*	0.591285	0.558949	0.624309	0.56179
*c*	0.784373	0.676604	0.851618	0.699287
*A*	0.691961	0.705747	0.734807	0.71341
*B*	0.509391	0.642045	0.574586	0.689394
*C*	0.606311	0.592511	0.59116	0.549927

In the analysis of the necessary condition by fsQCA, “Consistency” expresses the degree that the empirical data is in line with a postulated subset relation [48, 50]. It is generally believed if a condition’s Consistency value is higher than 0.9, it can be deemed as the necessary condition for the outcome. Results show that all condition’s Consistency value (i.e., condition A, B, C, a, b, c) are less than 0.9, which means each condition alone is not able to obtain high performance, i.e., any single cooperation cannot lead to “high economic performance” or “high social/environmental performance”. Therefore, they will not be excluded from Truth table operations.

To sum up, this analysis further demonstrates that neither the trinity federation nor the trinity co-operative can achieve high economic or social/environmental performance by relying only on single cooperation of production, supply & sale, or credit. That is to say, the trinity federation and the trinity co-operative need to adopt diversified collaborative patterns to achieve high performance. The analysis of the necessary condition is shown in Table 4.

#### 4.4.2 The analysis of sufficiency

Schneider and Wagemann [48] point out that sufficient conditions refer to the combination of conditions that can produce high performance. As long as the sufficient condition appears, the high performance can be achieved.

According to the criteria of outcome variables mentioned above, the high performance of cooperation can be divided into high economic performance and high social/environmental performance. And the situation of high economic performance is discussed first. In this study, the truth table was constructed by fsQCA3.0 software, and “Quine-McCluskey algorithm” was used for statistical analysis.

Based on the QCA rules, the sample’s frequency threshold value should be higher than “1”. Therefore, the sample’s frequency threshold value is set to 1.2 in the calculation of the truth table in this study. At the same time, the Consistency threshold was set to 0.7, which conforms to the corresponding operation rules of the fsQCA method. Schneider and Wagemann [48] pointed out that researchers should pay great attention to the background, characteristics and subjects of the study when selecting the exact location of Consistency threshold. Since trinity co-operatives and federations are in the latest trial phase, limited theoretical expectations could be obtained in the previous literature and many standards are set empirically. Therefore we should not arbitrarily choose the Consistency threshold by referring to some commonly accepted conventional routines. Instead, a consistency threshold value of 0.7 could include more possible collaborative patterns for agricultural co-operatives in the pilot phase to make their own location-specific choices. Based on the standards above, the corresponding Conservative solution term and Most parsimonious solution term are obtained by running the program of “Standard Analysis”, and Core Condition and Peripheral Condition can obtain the specific path of high economic performance.

#### 4.4.3 Economic performance

Table 5 summarizes the results of QCA analysis on economic performance, which shows that trinity cooperation can achieve high economic performance through three paths (i.e., collaborative patterns). The overall “Coverage” of this analysis is 75%. That is to say, the results explain 75% of cases of high economic performance. (sufficiency Coverage expresses how much of the outcome is covered by the sufficient condition). For a specific collaborative pattern, Consistency can be viewed as the pattern’s internal validity. The overall solution Consistency indicates to what degree the empirical data is in line with the overall solution, and the overall solution Coverage means the percentage of all cases’ set membership sores in the outcome covered by the solution term. Statistical analysis results show that the overall path consistency value is about 0.81. The three paths (collaborative patterns) are listed in Table 6. Due to the low value of collaborative pattern 3, special attention should be paid to its original case in the analysis [48].

**Table 5:** The paths (collaborative patterns) to “high economic performance”.

Model of high economic performance		Paths (collaborative patterns)		
		1	2	3
**Condition variables**	*a*	●	●	●
	*b*	●	◎	●
	*c*		●	●
	*A*			
	*B*		●	◎
	*C*	●	◎	
Consistency		0.72	0.83	0.63
Raw coverage		0.37	0.50	0.46
Unique coverage		0.13	0.18	0.15
Overall solution consistency		0.81		
Overall solution coverage		0.75		

**PS:** “●” and “◎” respectively represent the existence and non-existence of a condition. The blank space means that the condition can exist or not exist. The Core Conditions are marked with the capital letter “●” and “◎”; The Peripheral Conditions are marked with lower case “●” and “◎”.

**Table 6:** The specific analysis of collaborative patterns to “high economic performance”.

Collaborative patterns (paths)	Specific explanation
Collaborative patterns 1 (a*b*C)	This pattern shows that the agricultural co-operative can achieve high economic performance when production cooperation and supply & sale cooperation are implemented within the trinity co-operative, and credit cooperation is implemented by the trinity federation. Financing support is a prerequisite for agricultural production, and the trinity federation carries out credit cooperation to help the trinity co-operative better cooperates in production, supply & sale. The realization of this path is inseparable from the efforts of the co-operatives themselves, and the help of trinity federations. In general, the co-operative can achieves high economic performance when it plays a leading role in production, supply & sale, and continues to develop and improve itself with the necessary financial assistance form the trinity federation.
Collaborative patterns 2 (a*~b*B*C)	This pattern shows that high economic performance can be achieved when production cooperation is implemented within the trinity co-operative, as well as supply & sale, and credit cooperation implemented by the trinity federation. In this path, the trinity co-operative is usually a government-led co-operative. That is to say, under the high intervention of the trinity federation, the co-operative is only responsible for organizing the production activities (supply & sale cooperation particularly does not require the co-operative’ intervention), and the federation coordinates resources to provide supply, sale, and credit services. The realization of this path is closely related to the efforts of the trinity federation.
Collaborative patterns 3 (a*b*c*~B*)	This pattern shows that if the trinity co-operative has production, supply & sale, and credit cooperation, it can achieve economic performance without the help from the trinity federation (supply & sale cooperation particularly does not require federation’s intervention). A typical case of this pattern is Feng-yang trinity co-operative in Anhui province and its corresponding trinity federation. The economic benefits are well realized with the market vitality and internal information exchange within the trinity co-operative. Especially, this model needs the co-operative leader to have a lot of personal experience and skill. Feng-yang trinity co-operative has established its own brand, and its agricultural products have an edge on others in the local market due to the strong executive ability and market insight of the co-operative leader. This kind of trinity co-operatives can well adapt to market demand through internal information exchange and achieve high economic performance even without the trinity federations’ help. Also, there is no excessive intervention of the federation like inefficient government allocation and waste of resources. The implementation of this path particularly requires the ability of the co-operative leader, which may affect the accuracy of its Calibration leading to its low Consistency value.

According to the results above, we can find that capable agricultural co-operatives need complete loans and insurance guarantee. Credit cooperation is an important condition variable to achieve high economic performance. Such result confirms that financial integration has important practical significance though it is included in the traditional ASCI theory.

#### 4.4.4 Social/environmental performance

According to the classification of the outcome variables mentioned above, Table 7 is obtained when high social/environmental performance is set as the outcome variable.

**Table 7:** The paths (collaborative patterns) to “high social/environmental performance”.

Model of high economic performance		Paths (collaborative patterns)		
		1	2	3
**Condition variables**	*a*	●	●	●
	*b*	●		
	*c*		●	
	*A*	◎		◎
	*B*		●	●
	*C*			
Consistency		0.72	0.75	0.63
Raw coverage		0.51	0.53	0.36
Unique coverage		0.06	0.09	0.15
Overall solution consistency		0.70		
Overall solution coverage		0.68		

Thus, it can be seen that there are three paths to achieve social/environmental performance. The overall “Coverage” is 68%, which is to say the results explain 68% of the cases of high social/environmental performance. Also, the overall solution Consistency is 0.7, and this consistency value is affected by the extreme value of the third path (collaborative pattern 3). So we need to specifically analyze it when we need to adopt path 3 [51]. Strictly speaking, we cannot directly believe that these three paths can achieve high social/environmental performance, but we can still try to analyze these three collaborative patterns by empirical evidence (Table 8).

**Table 8:** The specific analysis of collaborative patterns to “high social/environmental performance”.

Collaborative patterns (paths)	Specific explanation
Collaborative pattern 1 (a*b*~A)	This path shows that high social/environmental performance might be achieved by production, supply & sale cooperation of the trinity co-operative, while the trinity federation should not imply production cooperation. In this pattern, the trinity co-operative estimates the competitive agricultural products on the market by measuring the previous sales data, and conveys them to the production department through direct information exchange. The production department can introduce new varieties to promote innovative technologies, improving product quality and food safety, reducing agricultural pollution and rot caused by unsold agricultural products through timely internal communication.
Collaborative pattern 2(a*c*B)	This path shows that high social/environmental performance might be achieved by production and credit cooperation of the trinity co-operative, and supply & sale cooperation of the trinity federation. In this pattern, the trinity federation uses government power to collaborate with external organizations to provide the trinity co-operative with steady suppliers and customers. Also, the trinity co-operative can improve product quality and ensure food safety through production and credit cooperation. This is a model of achieving social/environmental performance by stable sales channels.
Collaborative pattern 3 (a*~A*B)	This path shows that high social/environmental performance might be achieved by production cooperation of the trinity co-operative, and supply & sale cooperation of trinity federations, while the trinity federation should not imply production cooperation. A typical case of this pattern is Bo-hai trinity co-operative in Beijing and its corresponding trinity federation. This trinity co-operative has a solid advantage in the production aspect. Bo-hai trinity co-operative has a leading position with several national patents in China, and its products are well known not only locally but also nationally. Due to its substantial advantages in production, it seems that it only needs trinity federation’s external promotion and no other internal cooperation to achieve high social/environmental performance, such as attracting co-operative members and improving product safety and quality. However, such a trinity co-operative with advanced national technology is not common in China, and the reference of this collaborative pattern needs more careful investigation.

Although there is no guarantee that every path can achieve high social/environmental performance significantly, we can find that supply & sale cooperation is the core conditional variable. In order to achieve high social/environmental performance and improve product quality and safety, the trinity federation needs to establish a strong and effective co-operative relationship with external partners, while the trinity co-operative needs to obtain timely information on supply & sale then strengthening information exchange between departments.

## 5 Conclusion and limitations

To answer the first research question (Why is it possible for agricultural co-operatives to participate in ASCI?), starting from the nature and structure of agricultural co-operatives, we prove that trinity co-operatives could complete internal integration through information communication among production, supply & sale, and credit departments, and trinity federations could carry out external integration through cooperation with other partners in ASC in terms of production, supply & sale, and credit. For the second question, we have constructed a typology framework of agricultural co-operatives in ASCI and have explained its operation process. On the third research question answer, six collaborative patterns have been proposed based on the study of QCA. Three collaborative patterns (paths) can be obtained to achieve high economic performance along with another three paths obtained on high social/environmental performance.

In general, this study provides a complete answer to the question that how should agricultural co-operatives act in ASCI. We have made many theoretical contributions. First of all, our research has completed the ASCI literature by studying ASCI from the perspective of Chinese agricultural co-operatives. This study further emphasizes that ASCI concept differs greatly from the SCM concept [19, 20]. The trinity co-operatives’ internal information integration and the trinity federations’ cooperation with partners (especially logistics companies) in ASCI have specific differences with SCM. Additionally, social/environmental performance in ASCI, such as product quality, food safety, agricultural pollution and rot reduction also have practical significance impacts that SCM does not have. Second, we have contributed to the agricultural co-operative management theory by proposing an innovative framework (Fig 3) that explores the role of trinity co-operatives and trinity federations separately and mutually for the first time. Third, we have proposed six collaborative patterns based on 26 latest agricultural co-operatives to figure out the detailed process of co-operatives’ participation in ASCI.

This research has suggestions and implications for further practical action and policymaking. First of all, Chinese policy-makers can use the theoretical framework and the collaborative patterns to customize different policies for diverse trinity co-operatives and trinity federations. The direct application of these six specific collaborative patterns can only be carried out in China, because trinity co-operatives and trinity federations are unique agricultural organizations in China. For those co-operatives in different regions that do not have trinity federations as governmental assistance, they can learn from the experience of the Huangzhang-Planting Agricultural Co-operative, the Hezhou Turtle Co-operative and Arla Foods in Section 3.1, and develop the competitiveness to achieve external integration in ASC.

Secondly, apart from China, other countries can also get fruitful guidance from these six collaborative patterns. The results of QCA analysis show that credit cooperation is an important element to achieve high economic performance, and supply & sale cooperation is the core element to achieve high social/environmental performance. We offer the local governments and leaders of co-operatives a guide on how to make their co-operatives participate in ASCI and achieve high performance. For example, for some co-operatives with similarities in Malaysia [41], more attention can be paid to strengthening credit cooperation such as improving agricultural loan and insurance policies to achieve high economic performance rather than just keeping eyes on technology and logistics. In addition, for some co-operatives in Denmark and South Korea which need to achieve high social/environmental performance [15], supply & sale cooperation may have unexpected gains compared with inefficient government subsidies.

Thirdly, more emphasis should be laid on the financial integration of agricultural co-operatives. Many capable co-operatives have shown exceptional ability in credit cooperation, which is vital in the further development of ASCI. Within co-operatives, credit cooperation is based on production cooperation and supply & sale cooperation, and the degree of information asymmetry among members is relatively low. In such organizations, the implementation of financial management and credit rating is more convenient and efficient. Agricultural co-operatives should take advantage of this to increase financial support and agricultural insurance for farmers. Besides, as organizations that unite the power of farmers, co-operatives can conduct financial cooperation with other ASC partners from a higher position, promoting ARIF (accounts receivable and inventory financing), policy-based agricultural loans and insurance with leading enterprises, such as commercial banks, insurance companies, agricultural material companies, processing companies, electric business platforms and wholesalers.

Fourthly, the social/environmental performance of agricultural co-operatives in ASCI enables co-operatives to achieve sustainable development while gaining economic benefits. Such an environment-friendly production process is now advocated all over the world and can be studied by China and many other countries.

The study is unlikely to be without limitations. Firstly, we propose the framework and patterns by studying 26 latest agricultural co-operatives and combing various literature. The results of the study should be empirically tested by other methods. Secondly, the cases in the study are trinity co-operatives and trinity federations, which are at an early stage of development. Researchers need to pay attention to three issues when using the findings: (1) The cases we use are the latest agricultural co-operatives and many traditional co-operatives in China are not included in the case selection. Therefore, it is questionable whether the research results are applicable to traditional co-operatives or not. (2) In the study, some characteristics of the cases may be specific to China, such as the personal charm of trinity co-operative leaders and the financial support from trinity federations. Readers should scrupulously extend this result to other institutional contexts. (3) Due to the number of the conditional variables, there are 64 possible co-operative patterns in the mathematical logic, which leads to the arithmetic remainders [48] that the number of truth table rows simply outnumbers the cases at hand. Therefore, future studies can add the samples and comprehensively consider and test the universality of the research results.
